# Transforming growth factor-β1 signaling promotes epithelial-mesenchymal transition-like phenomena, cell motility, and cell invasion in synovial sarcoma cells

**DOI:** 10.1371/journal.pone.0182680

**Published:** 2017-08-22

**Authors:** Yan Qi, Ning Wang, Yonglai He, Jun Zhang, Hong Zou, Wenjie Zhang, Wenyi Gu, Yalan Huang, Xiaojuan Lian, Jianming Hu, Jin Zhao, Xiaobin Cui, Lijuan Pang, Feng Li

**Affiliations:** 1 Department of Pathology and the Laboratory of Xinjiang Endemic and Ethnic Diseases, Shihezi University School of Medicine, Shihezi, Xinjiang, China; 2 Department of ICU Intensive Care, the First Affiliated Hospital, Shihezi University School of Medicine, Shihezi, Xinjiang, China; 3 Department of Medical Genetics, Shihezi University School of Medicine, Shihezi, Xinjiang, China; 4 Australian Institute for Bioengineering and Nanotechnology (AIBN), University of Queensland (UQ), St Lucia, Brisbane, Australia; 5 Department of Tumor Blood, Jiangjin Central Hospital of Chongqing, Chongqing, P.R. China; 6 Department of Pathology, Beijing Chaoyang Hospital, Capital Medical University, Beijing, China; University of South Alabama Mitchell Cancer Institute, UNITED STATES

## Abstract

The epithelial-to-mesenchymal transition (EMT) and the reverse process (the mesenchymal-to-epithelial transition [MET]) have been shown to be associated with tumor cell invasion and metastasis in different carcinomas. The EMT and MET have recently been shown to play a key role in the pathogenic processes of sarcomas, which are completely different from those of carcinomas. However, the definitive roles of the EMT in the tumorigenesis of synovial sarcomas remain unknown. Here, we explored whether transforming growth factor (TGF)-β signaling, an important oncogenic event in synovial sarcoma, modulates tumor cell characteristics related to the EMT, such as cell adhesion, migration, invasion, and proliferation. Interestingly, we found that TGF-β1 induced tumor cell activation, resulting in a tendency to aggregate and biphasic-like features. TGF-β1 also caused downregulation of E-cadherin and subsequent upregulation of N-cadherin, Snail, and Slug, which are responsible for EMT-like phenomena and increased cell motility and invasion. To further investigate the roles of TGF-β1 in the EMT, we established a SW982 cell line with stable TGF-β1 inhibition viaSB431542.These cells exhibited significantly decreased motility, migration, and proliferation (*P* = 0.001). Taken together, our data demonstrated that alterations in the TGF-β1/Smad signaling pathway could regulate the expression of EMT-related factors and the EMT process, resulting in changes in tumor cell invasion, migration, and proliferation in synovial sarcoma cells. These results may provide a important insights into therapeutic interventions and contribute to the present understanding of tumor progression in patients.

## Introduction

Sarcomas are uncommon yet diverse mesenchymal malignancies. Synovial sarcoma (SS) is a type of mesenchymal tumor that exhibits typical epithelial differentiation in terms of morphology and immunohistochemical phenotype. Additionally, SS is generally characterized as a high-grade and aggressive soft tissue sarcoma that is most commonly observed in the extremities of young adults[1].Patients with SS have a poor prognosis(10-year survival rate: 10–30%)[1].Moreover, SS is associated with a specific t(x;18)(p11.2;q11.2) chromosomal translocation that generates the *SYT-SSX* fusion gene (involving SSX1, SSX2, orSSX4); this gene is a potent oncogene that plays a key role in the pathogenesis of SS. Kawai et al.[2]first described relationship between the *SYT-SSX* fusion transcript and the histologic subtype and clinical behaviors of SS. Moreover, Saito et al. suggested that almost all biphasic SS (presence of glandular epithelial differentiation with lumen formation) harbors the *SYT-SSX1* fusion gene [3].

Recently, sarcomas have been shown to have an epithelial-like in phenotype, which is closely related to the epithelial-to-mesenchymal transition (EMT) and its reverse process, the mesenchymal-to-epithelial transition (MET)[4, 5]. Studies in the past 20 years have shown that the EMT is associated with various cancer-related processes, including metastasis and induction of tumor progression/metastasis in several types of sarcoma[5].Various transcription factors or secreted components, including fibroblast growth factor, hepatocyte growth factor, transforming growth factor-β1 (TGF-β1), and β-catenin[6]can contribute to or are required for the EMT/MET. However, the specific role of TGF-β1 in the EMT/MET in SS is not yet clear.

SS is thought to be a useful model for investigating the potential mechanisms involved in the aberrant EMT/MET in mesenchymal neoplasms because of the possibility of biphasic differentiation. Thus, in the present study, we investigated the role of TGF-β1 in EMT-like phenomena and cell motility in SW982 synovial sarcoma cells. We also investigated the potential mechanisms underlying this process. Our data showed that the TGF-β1 pathway induced tumor cell invasion, migration and proliferation in SS.

## Materials and methods

### Cell culture, antibodies, and reagents

SW982 human synovial sarcoma cells were purchased from the Shanghai Institute of Biochemistry and Cell Biology (ATCC, HTB-93™).The SW982 cell line was derived from a biphasic SS removed from a 25 year old woman as previously described[7].Cells were grown in L-15 medium containing 10% fetal bovine serum (FBS; Gibco, CA, USA),100 U/mL penicillin, and 100 mg/mL streptomycin (Invitrogen, Karlsruhe, Germany) at 37°C in a normal humidified atmosphere.

### Cellular treatment methods

To induce the EMT, cells were seeded in 6-well plates and grown to 70–80% confluence in complete growth medium. Recombinant human TGF-β1 (R&D Systems, MN, USA) was reconstituted in 4mMHCl containing 0.1% bovine serum albumin. Cells were then incubated in serum-free medium supplemented with TGF-β1 at concentrations of 0, 1, 5,or 10ng/mL at 37°C in a normal humidified atmosphere and were then harvested 36 h after treatment. MTT assays were used to evaluate cell proliferation. All experiments were performed in triplicate and repeated three times.

To inhibit the EMT, SB431542 (Sigma Systems, FL, USA), a TGF-β1inhibitor, was dissolved at a concentration of 10 mM in dimethylsulfoxide (DMSO). Cells were incubated in serum-containing medium supplemented with 0, 1, 5, or 10 μM SB431542at 37°C in a normal humidified atmosphere and were then harvested at 24 h after treatment. All experiments were performed in triplicate and repeated three times.

### Real-time quantitative reverse transcription-polymerase chain reaction (RT-qPCR)

mRNAs of *E-cadherin*, *N-cadherin*, *Snail*, *Slug*, *Smad2*, *Smad3*, *and TGF-β1* were quantified using reverse transcription real-time quantitative polymerase chain reaction (RT-qPCR) on an ABI PRISM 7500 Fast Sequence Detector (Applied Biosystems, Foster City, CA, USA). The cDNA was reverse transcribed from 1μg of total RNA using oligo (dT) primers according to the manufacturer’s protocol (Applied Biosystems). RT-qPCR was performed using SYBR Green master mix and the following primer sets: SS18-SSX forward 5′-CAGGGCTACGGTCCTTCAC-3′ and reverse 5′-TTCGTCCTCTGCTGGCTTC-3′; TGF-β1 forward 5′-TGCTAATGGTGGACCGCAA-3′ and reverse 5′-CACTGCTTCCCGAATGTCTGA-3′; Smad2 forward 5′-TCTCCGGCTGAACTGTCTCCTA-3′ and reverse 5′-GCGATTGAACACCAGAATGCA-3′; Smad3 forward 5′-ATGGAGCTCTGTGAGTTTGCCT-3′ and reverse 5′-GCGATTGAACACCAGAATGCA-3′; Slug forward 5′-ATGCATATTCGGACCCACACATTAC-3′ and reverse 5′-AGATTTGACCTGTCTGCAAATGCTC-3′; E-cadherin forward 5′-TGCTAATGGTGGACCGCAA-3′ and reverse 5′-TCCTATCTTGGGCAAAGCAACTG-3′; N-cadherin forward 5′-CGAATGGATGAAAGACCCATCC-3′ and reverse 5′-GGAGCCACTGCCTTCATAGTCAA-3′; Snail forward 5′-GACCACTATGCCGCGCTCTT-3′ and reverse 5′-TCGCTGTAGTTAGGCTTCCGATT-3′; and β-actin forward 5′-GAGCGGGAAATCGTCCGTGACATT-3′ and reverse 5′-GATGGAGTTGAAGGTAGTTTCGTG-3′. Reactions were performed in a 96-well spectrofluorometer using an Rmalcycler (Applied Biosystems) under the following conditions: 5 min at 50°C, followed by 40 cycles of 10 s at 95°C and 30 s at 60°C. Relative transcript levels were calculated using the 2^-ΔΔCt^ method, as specified by the manufacturer.

### Immunofluorescence imaging

Approximately 3 × 10^4^L-15 cells were plated on fibronectin-coated (10 μg/mL) cover slips in 12-well plates. Cells were treated with TGF-β1 with or without SB431542 for 36 h, fixed in 3.7% formaldehyde for 15 min, and permeabilized in 0.2% Triton X-100/phosphate-buffered saline (PBS) for 5 min. Cells were then incubated with rabbit anti-TGF-β1 (1:200 dilution; Abcam, Cambridge, UK) for 12h at 4°C. Following washing with PBS, cells were incubated with secondary antibodies for 30 min at 37°C. Alternatively, cells were incubated with mouse anti-E-cadherin (1:100 dilution; Abcam) for 12h at 4°C, washed in PBS, and incubated with secondary antibodies for 30 min at 37°C. Finally, cells were incubated with 4′,6-diamidino-2-phenylindole (DAPI; 1:600 dilution) for 1 h at 37°C, washed in PBS, and mounted in Mowiol medium. Cell images were captured using a fluorescence microscope.

### Cell migration and invasion assays

Wound-healing assays were performed to test cell migration. SW982 cells (5 × 10^5^/mL) were plated in 6-well plates and grown overnight to near confluence. A wound was created in the cell monolayer, and cells were then washed with PBS and incubated in serum-free L-15 medium. During incubation, cells were treated with different concentrations of TGF-β1 with or withoutSB431542. Images were acquired at6, 12, and 24 h, and data were analyzed statistically.

For invasion assays, we used transwell chambers (8μm, 24-well format; Corning Co., USA) or Matrigel-coated transwell chambers (BD Bioscience, USA), inserted into 24-well cell culture plates. Cells (3 × 10^4^ in 0.2 mL of serum-free medium) were added in the upper chamber, while 0.6 mL of L-15 medium containing 20% FBS was added to the lower chamber. These cells were cultured for 24 h, and cells that had migrated or invaded through the inserts were then fixed in methanol for 20 min, stained with crystal violet, and counted in three random fields under a microscope (Olympus BX3, Japan) at magnification of 40×, 100×, or 200×.

### Cell Counting Kit 8 (CCK8) proliferation assays

Cells were seeded in 96-well plates at a density of 3 × 10^3^cells/well and then cultured for 24 h in serum-free L-15 medium. The cells were then treated with different concentrations of TGF-β1 and SB431542. The cells were counted every 24 h for 3 days. Before counting, the cells were evaluated using CCK-8 assays (Dojindo, Kumamoto, Japan). The absorbance at 450 nm was determined with a microplate reader (Bio-Rad, Hercules, CA, USA).

### Statistical analysis

We used a factorial design, and all experiments were repeated three times. The results are presented as the mean ± standard deviation. All data obtained were analyzed using SPSS software, version 18.0 (SPSS, Inc., Chicago, IL, USA). The data met the criteria for independence, normality, and homogeneity of variance; therefore, a reference sample t-test was selected for analysis. *P*<0.05 was considered statistically significant.

## Results

### Recombinant human TGF-β1 induced an EMT-like process in SW982 cells

**Fig 1:**
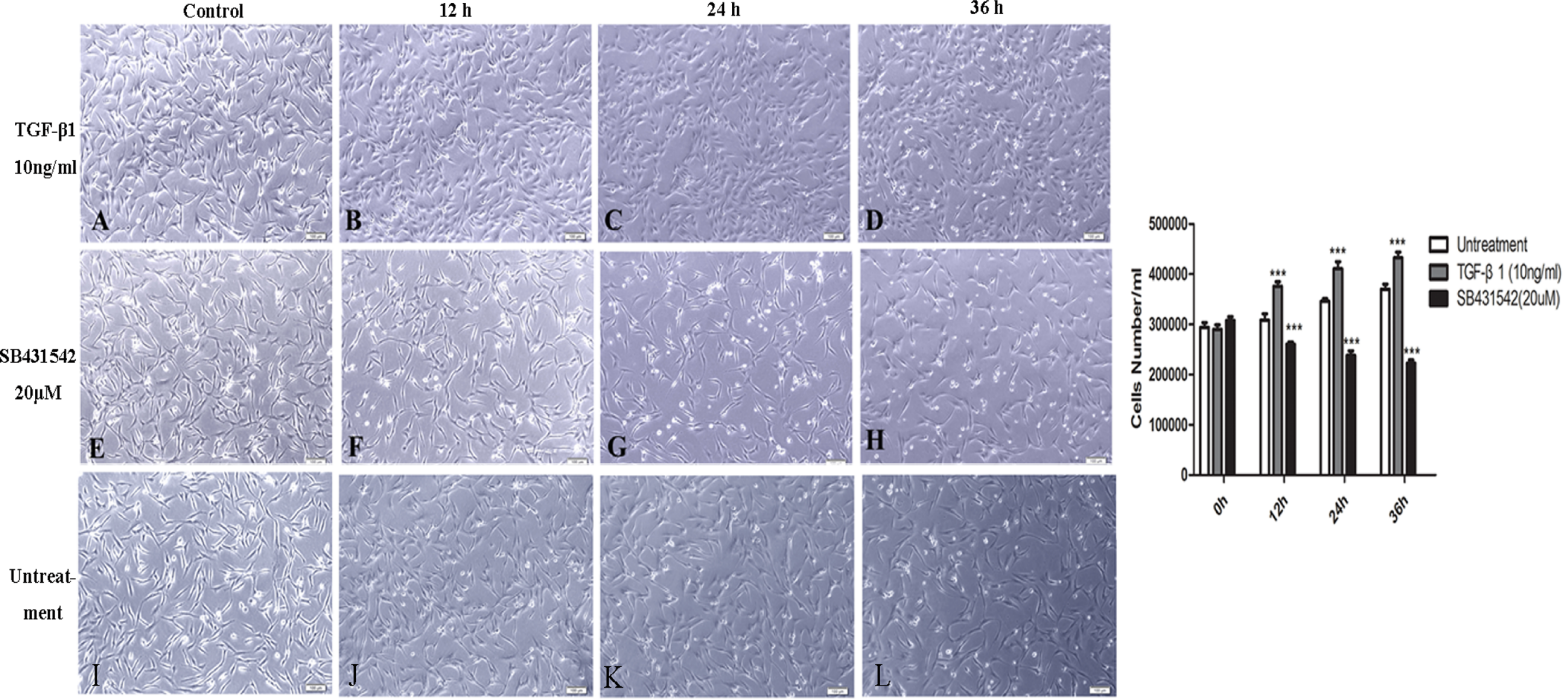
Morphological changes in SW982 cells after TGF-β1 or SB4315425 treatment. A, E. Untreated SW982 cells, 100×; B–D. SW982 cells treated with 10 ng/mL TGF-β1 for 12, 24, or 36 h, 100×; F–H. SW982 cells were treated with the inhibitors SB4315425 (5 mM) for 12, 24, or 36 h, 100×.

To determine the effects of TGF-β1 on the activity of SW982 cells, we treated the cells with 0, 1, 5, or 10ng/mL TGF-β1. As shown in Fig 1, TGF-β1 treatment increased the cell number and activity in a concentration-dependent manner (Fig 1A–1D) at 0, 12, 24, and 36h, and the cells tended to have a biphasic morphology. Compared with the control, we concluded that the most suitable concentration was 10ng/mL(*P*< 0.01). Interestingly, TGF-β1 treatment caused a MET-like cell morphology with increased cell-cell contact and more cells showing a short spindle-like shape and congregated growth (Fig 1B–1D). Conversely, after treatment with the inhibitor SB431542, the number and activity of cells were also significantly decreased in a concentration-dependent manner (Fig 1E–1H), particular at a concentration of 20nM(*P*< 0.01). AndSB431542 also can suppressed the effects of TGF-β1 to some degree (S1 Fig). These phenomena indicated that the TGF-β pathway may play an important role in the progression of SS.

### TGF-β1 regulated the expression of various EMT-related markers in SW982 cells

**Fig 2:**
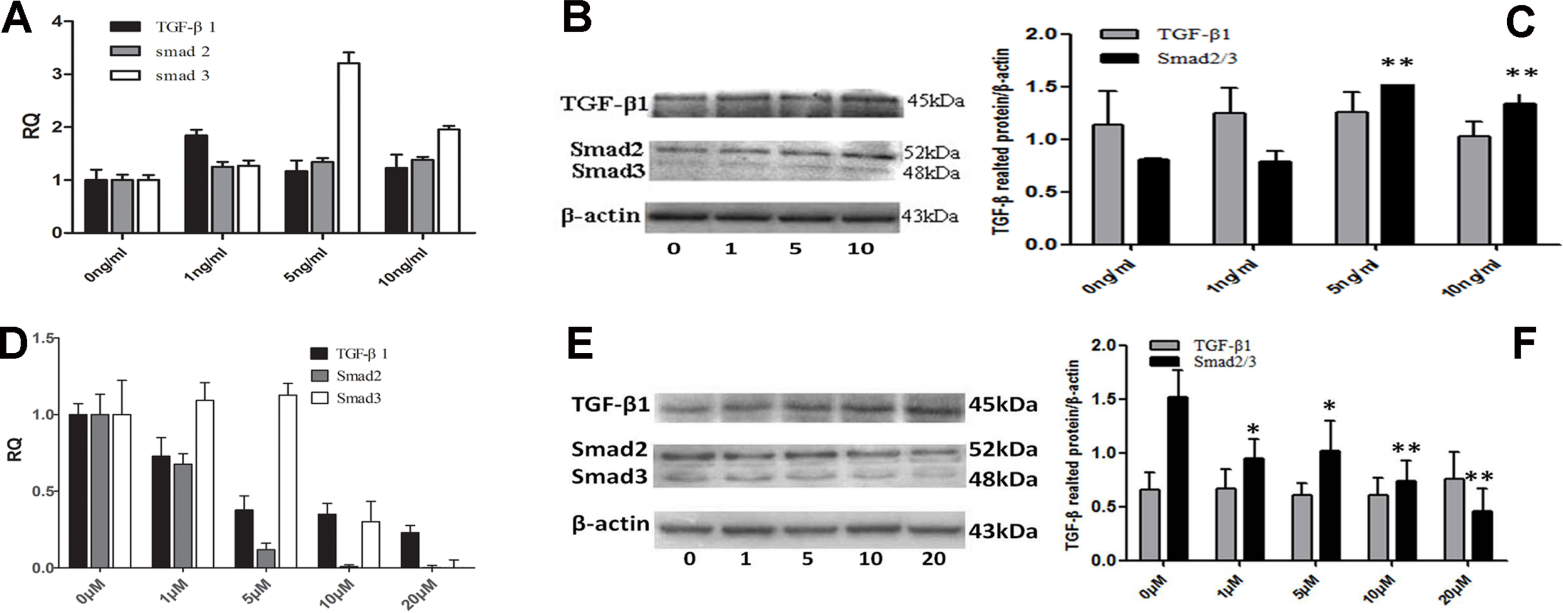
TGF-β signaling in synovial sarcoma. A. RNA was isolated from SW982 cell samples after treatment with1, 5, or 10ng/mL TGF-β1, and relative mRNA levels were quantified using RT-qPCR. Error bars represent the standard error of the mean. B, C. Levels of phosphor-Smad2/3 after TGF-β1 treatment. D. Cells were serum-starved and treated with the TGF-β1 inhibitor SB431542for 24 h, andrelative mRNA levels were evaluated. E, F. After inhibition of TGF-β1 by SB431542, the levels of phospho-Smad2/3 were evaluated. *, P < 0.05. Error bars represent standard deviations (SDs), except where indicated.

To examine the activation status of the TGF-β/Smad pathway, we detected the TGF-β and Smad2/3 levels. In SW982 cells, expression of Smad2/3 was induced by recombinant human TGF-β1. Our data showed that the mRNA and protein levels of Smad2 were prominently increased after1, 5, or 10 ng/mL TGF-β1 treatment (*P* = 0.002, 0.001, and 0.001, respectively); Smad3 levels were also upregulated (*P* = 0.025, 0.001, and 0.054 respectively; Fig 2A–2C). Conversely, SB431542inhibited the mRNA and protein expression of smad2(*P* = 0.001; Fig 2D–2F), and only slightly suppressed the expression of smad3.

**Fig 3:**
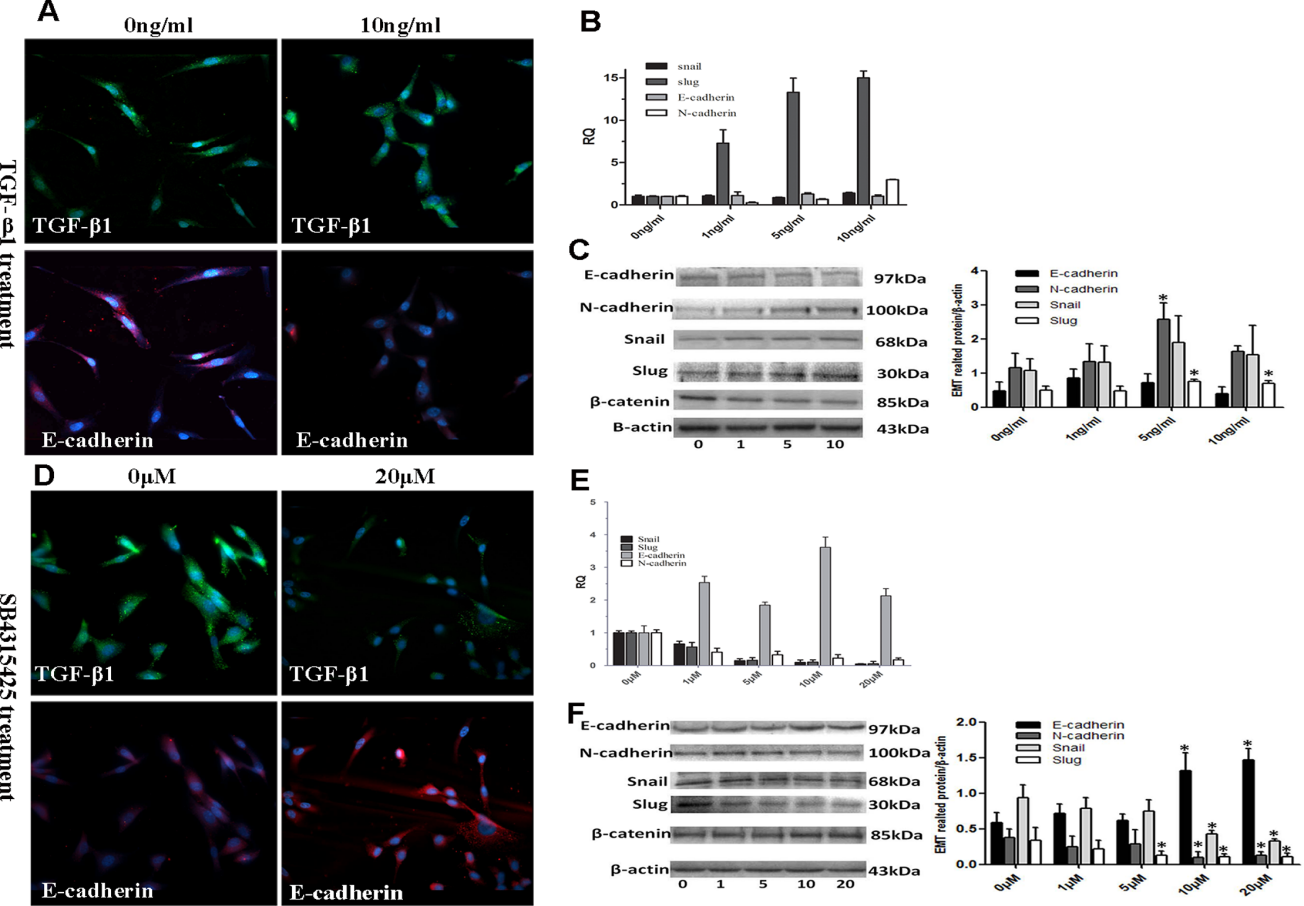
TGF-β1 induced the expression of EMT-related genes. A. Immunofluorescence staining showing E-cadherin expression following treatment with 10 ng/mL TGF-β1 for 36 h. B. RT-qPCR was used to evaluate changes in the levels of Snail, Slug, N-cadherin, and E-cadherin mRNA after TGF-β1 treatment. C. Western blot analysis of the targets in B, evaluated with and without TGF-β1 treatment. D. Western blot analysis of the targets in B, evaluated with and without SB431542 treatment (20μM)for 36h. E, F, effects of SB431542 treatment on the mRNA and protein levels of Snail, Slug, and N-cadherin.

To test whether TGF-β1 induced EMT in SS cells and to investigate the association between the TGF-β pathway and EMT-related markers, SW982 cells were treated with recombinant human TGF-β1 and SB431542. E-cadherin protein and mRNA levels were determined by RT-PCR, western-blot, and immunofluorescence staining. The results showed that the expression of E-cadherin was affected by the inhibitor SB431542 treatment in a concentration-dependent manner (Fig 3A, 3D and 3E) and was slightly decreased upon TGF-β1 treatment (Fig 3B and 3C).

Although E-cadherin was upregulated at lower concentrations, downregulation of E-cadherin was noted in the presence of 10ng/mL TGF-β1. In contrast, N-cadherin expression increased, particularly in the presence of 10ng/mL TGF-β1(*P* = 0.002). The transcription factor Snail was also significantly upregulated (*P* = 0.0001) in a concentration-dependent manner, whereas Slug expression was only slightly increased compared with that in the control (Fig 3B and 3C). To further confirm the essential role of TGF-β1 and its downstream targets in the EMT, we treated the cells with SB431542. Expression of the EMT-related marker E-cadherin increased(*P* = 0.001), whereas the expression of N-cadherin, Snail, and Slug decreased in a concentration-dependent manner (*P* < 0.05; Fig 3D–3F).

### TGF-β1 enhanced SS cell adhesion and motility

**Fig 4:**
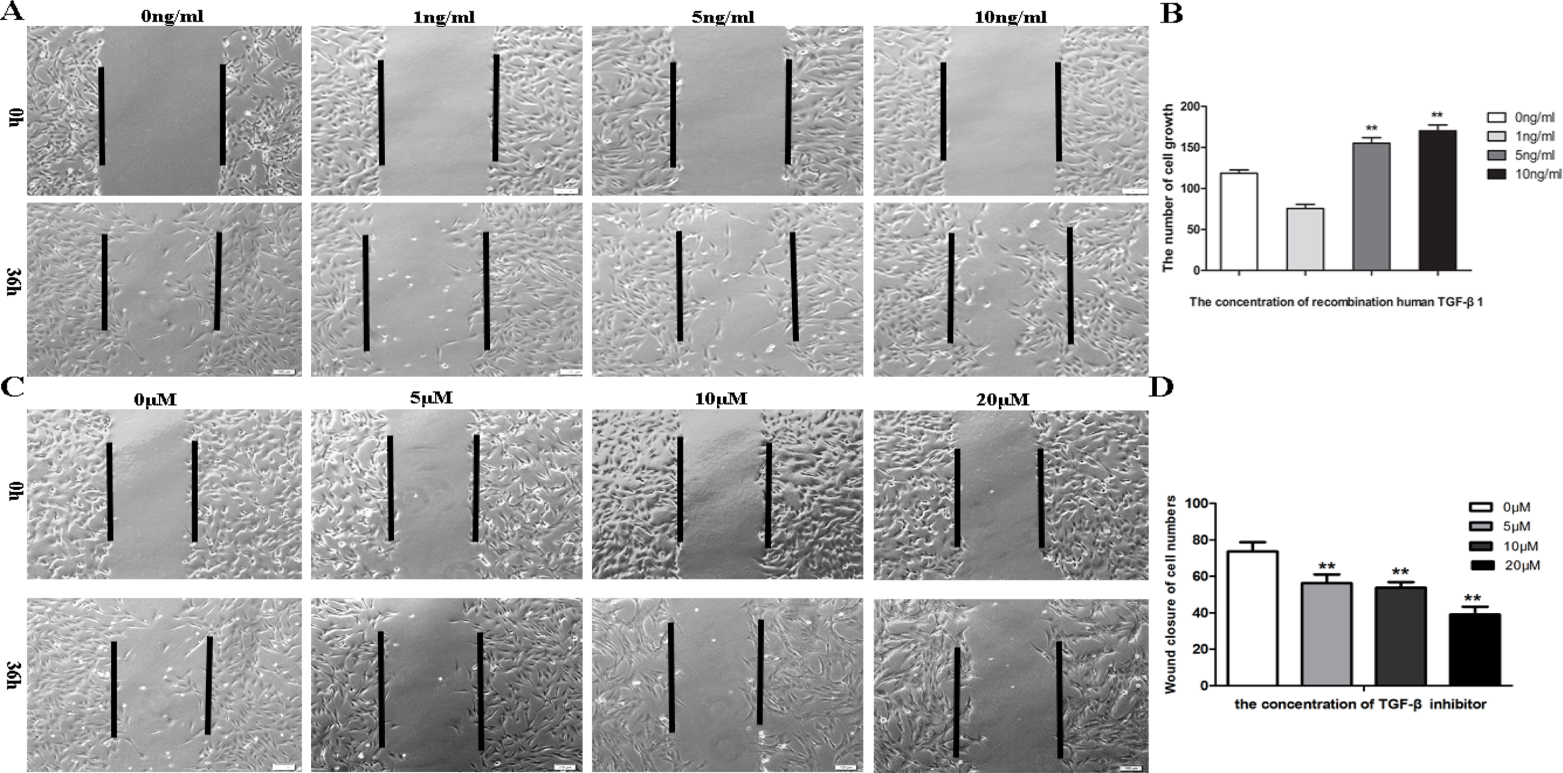
Effects of TGF-β1 and SB4315425 on the migration of SW982 cells. A–D. Wound healing assays showing the effects of TGF-β1 (A, B) and SB4315425 (C, D) on cell migration. Vertical lines indicate the wound margins. Experiments were performed three times with similar results.

Because TGF-β1 expression induced changes in the MET-like cell morphology and cell-cell contacts, we next examined whether TGF-β1could affect cell adhesion and motility, which are important properties of the EMT. We found that the migration ability of SW982 cells was poor, particularly in the absence of serum. However, SW982 cells adhered to culture plates more quickly and spread out faster compared with the control group when recombinant human TGF-β1 was added for 36 h. In wound-healing assays showed that TGF-β1-treated cells filled the wounded area faster than control groups (Fig 4A). Moreover, enumeration of adherent cells within the wound area indicated that the number of cells was significantly different at concentrations of up to 5 and 10ng/mL (*P* = 0.0001; Fig 4B). Conversely, wound-healing ability was blocked, and few cells migrated into the wound area in the presence of 10 or 20 μM SB431542 after 36 h (Fig 4C and 4D).

### TGF-β1 increased SW982 cell invasion and proliferation

**Fig 5:**
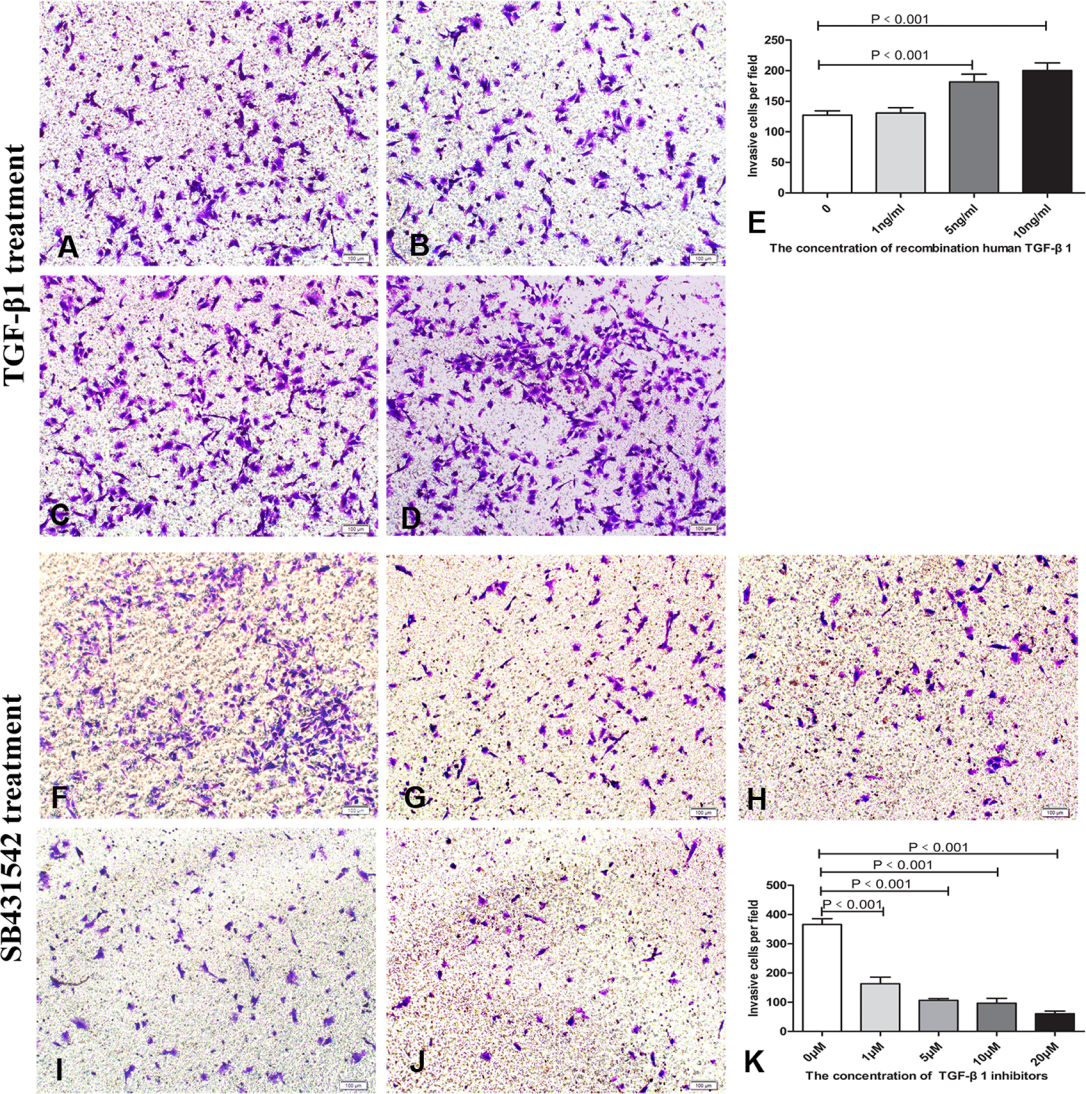
Effects of TGF-β on cell migration. A–E. Effects of TGF-β1 on the invasion of tumor cells (A-D: 0, 1, 5, 10ng/ml, respectively). F–K. Effects ofSB4315425 on the invasion of tumor cells. Similar results were observed in three independent experiments (F-J: 0, 1, 5, 10, 20μM, respectively).

**Fig 6:**
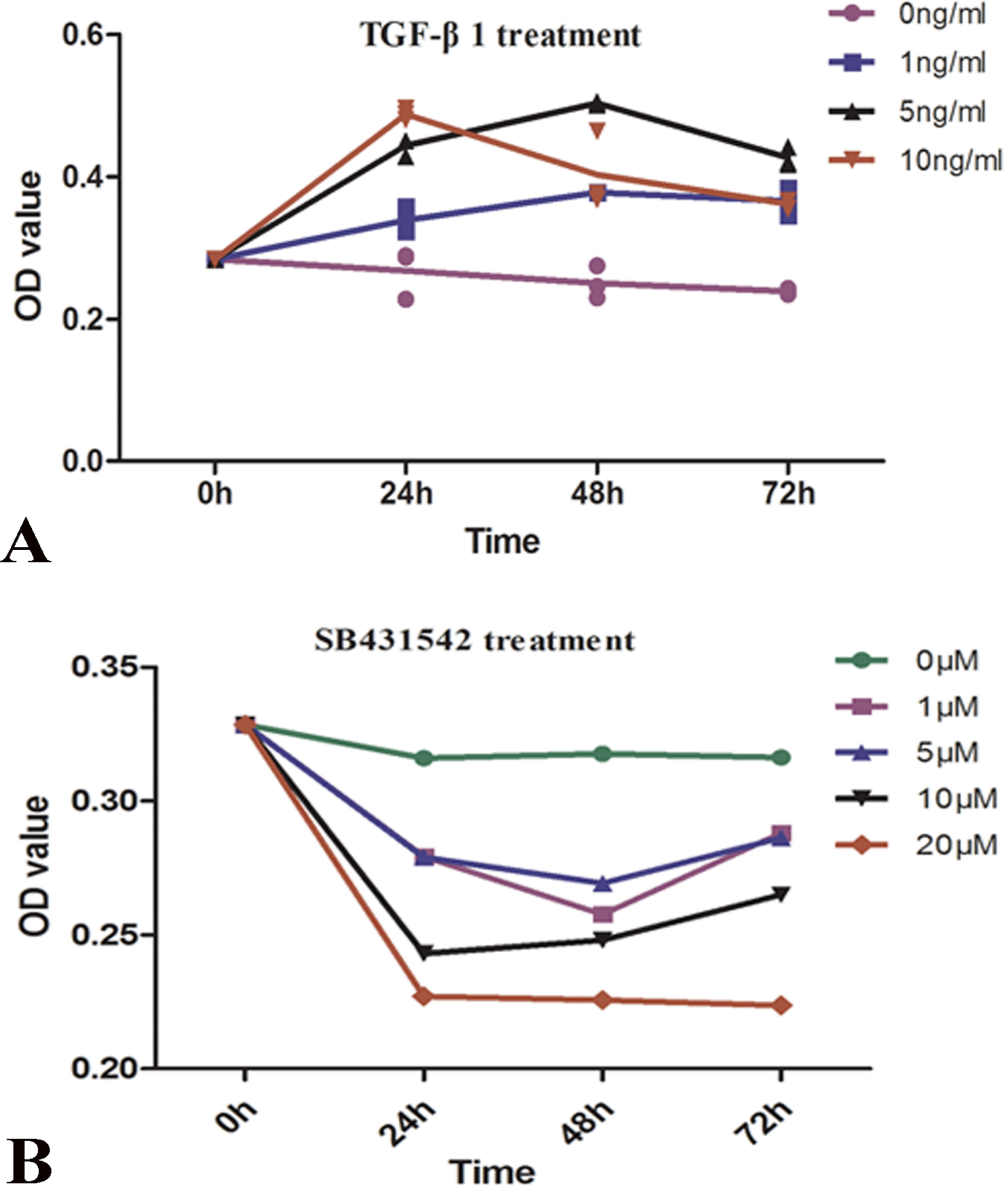
Effects of TGF-β on cell proliferation. CCK8 assays demonstrated that TGF-β1 induced cell proliferation(Fig 6A), ConverselySB431542 inhibited this increase in cell proliferation (Fig 6B).

Next, we assessed the effects of TGF-β1 on cell invasion and proliferation. Treatment with 5 or 10 ng/mL TGF-β1 significantly enhanced cell invasion compared with that in control groups (*P* = 0.0001; Fig 5A–5E). As expected, SB431542 efficiently suppressed cell invasion in a concentration-dependent manner (Fig 5F–5J). Similarly,CCK8 assays demonstrated that treatment with 5 ng/mL TGF-β1 for 48 h (Fig 6A), whereasSB431542 inhibited cell proliferation in a concentration-dependent manner (Fig 6B).

## Discussion

SS is a highly aggressive type of soft tissue sarcoma with typical dual epithelial and mesenchymal differentiation. More than 90% of SS samples harbor a specific fusion gene[2], which can influence the maintenance of tumorigenicity through regulation of various transcription factors, including Snail, Slug, ZEB, and Twist. Moreover, the progression of SS is closely related to the EMT[8, 9]. Several recent studies have shown that the MET promotes the growth of epithelial tumor cells at distant sites during metastasis[10–12]. Additionally, the MET has been shown to play a key role in biological and clinical processes in mesenchymal tumors and sarcomas. In particular, some types of sarcomas can exhibit epithelial differentiation(nested epithelial or glandular morphology), as evaluated by histopathology or based on the immunophenotype[8, 13, 14].

TGF-β1 has been well characterized as a critical modulator of the EMT in many types of carcinomas[15], but has not been frequently described in sarcomas. Pickup et al.[16] showed that TGFβ1is a major inducer of epithelial tumor progression through the EMT; this function as been well established in experimental models in various epithelial tumors. However, the roles of TGF-β1 in sarcomas, tumor stroma, and mesenchymal neoplasms are still unclear. Given the pleiotropic nature of TGF-β1, it is thought to be an important factor involved in regulating the development and progression of mesenchymal tumors.

Based on this background, in this study, we aimed to elucidate the role of TGF-β1in the EMT and the pathogenesis of SS. Our data showed that TGF-β1 induced tumor cell activation, promoting aggregation and biphasic features. Moreover, we showed that TGF-β1 induced tumor cell proliferation and invasion in SW982 cells. Blocking the TGF-β1 pathway inhibited these phenotypes. A previous study[17]showed that expression levels of TGF-β1 andSmad2/3 were high in both epithelial and spindle cell components of SS in tumor tissue. That study also showed thatSmad2/3 expression was correlated with the pTNM stage and that TGF-β1 exhibited a tendency toward positive correlation with pTNM stage. Thus, these results suggested that the TGF-β1/Smad signaling pathway is likely to play an important role in the progression of SS.

E-cadherin expression is a hallmark of EMT; reduced expression of E-cadherin is regulated by the expression of mesenchymal neural cadherin (N-cadherin) and vimentin, which alter tumor cell adhesion and motility[15], and thereby induce changes in morphology and invasive ability. The key transcription factors of the EMT, including Snail1,Snail2/Slug, ZEB1,ZEB2, and Twist, drive this reprogramming, which often results in a switch in cadherin expression[6]. Additionally, Snail is a prominent inducer of the EMT and strongly represses E-cadherin expression [18][19] showed that increased β-catenin transcription contributed to inhibition of epithelial differentiation, suggesting that β-catenin may drive the EMT in SS.

Machado[20]demonstrated that epithelial cell adhesion molecule and EMT markers are expressed in the Ewing’s sarcoma family of tumors and strongly recommended a prospective validation of immunomarkers with prognostic significance (e.g., desmoplakin, ZO-1, CK8/18, phospho-glycogen synthase kinase beta, and Snail) in a prospective series including localized and disseminated tumors. Cheng et al.[21]demonstrated that visfatin enhances the migration and invasion of osteosarcoma cells via the nuclear factor-kappaB/Snail-1/EMT pathway. Additionally, Dwivedi[22]found that TGF-β1 inducesSmad2/3 phosphorylation, migration, and EMT responses in uterine carcinosarcoma, a type of biphasic tumor. Our data showed that TGF-β1 and the inhibitor SB431542 promoted the E-cadherin and N-cadherin switch in SW982 cells. Expression of the transcription factor Snail was not observed; however, Slug expression was significantly increased by TGF-β1 treatment, facilitating the enhancement of cell invasion and proliferation. However, both Snail and Slug levels were decreased when TGF-β1 activity was blocked, and cell invasion and proliferation activities decreased. Thus, our results suggested that Slug and Snail promoted the EMT and augment the migration and invasion of SS cells in the context of the TGF-β1-induced EMT.

In conclusion, we demonstrated that activation of TGF-β1 may function as a main driver of the SS phenotype-switching model, characterized as an EMT-like conversion of proliferative and invasive SS cells. Specifically, we found that activation or inhibition of TGF-β1/Smad signaling regulated the expression of EMT-related factors in SS, thereby promoting or inhibiting the EMT process. Additionally, our results showed that the role of the transcription factor Slug was more obvious than that of Snail. Finally, TGF-β1 induced tumor cell invasion, migration, and proliferation in SS. We believe that this study provides support for future in vivo or clinical studies of targeted drug therapy.

## Supporting information

S1 Fig Cell growth effects induced by TGF-β1 and SB431542. Cells treated using TGF-β1 plus SB431542 showed cell growths more than cells treated with SB431542 alone but less than TGF-β1 alone (see S1 Fig A-D).
(TIF)
