# Supplementary figures and images for: Transforming growth factor-β1 signaling promotes epithelial-mesenchymal transition-like phenomena, cell motility, and cell invasion in synovial sarcoma cells
Source: PLoS One. 2017 Aug 22;12(8):e0182680. doi: 10.1371/journal.pone.0182680 (PMC5567493; doi:10.1371/journal.pone.0182680)

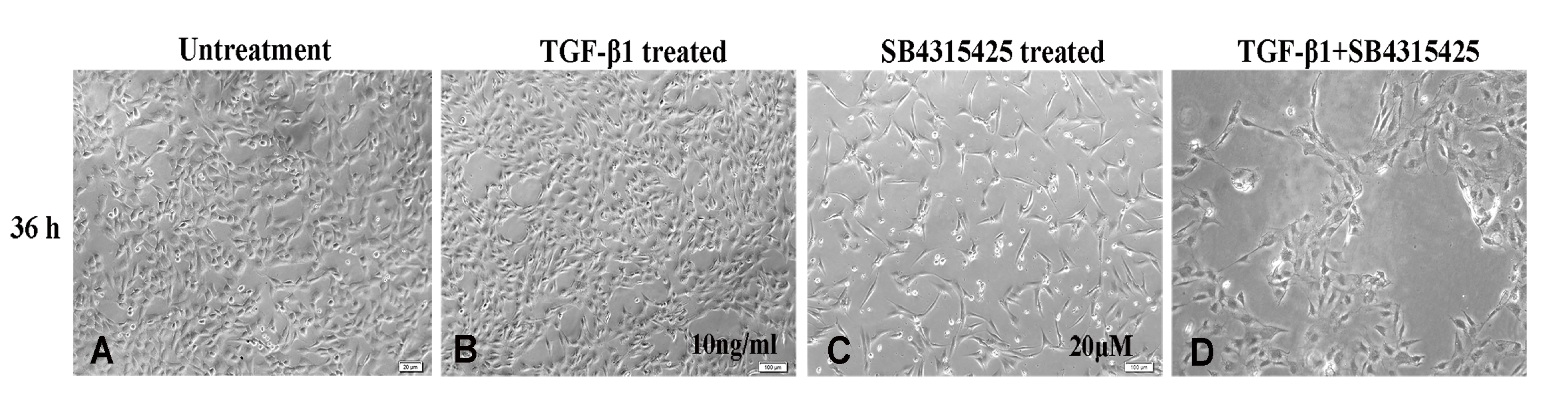

Supplement: S1 Fig — Cells treated using TGF-β1 plus SB431542 showed cell growths more than cells treated with SB431542 alone but less than TGF-β1 alone (see S1 Fig A-D). (TIF) [file pone.0182680.s001.tif]
